# A Remote Music-Based Service-Learning Project to Support Isolated Older Adults in Long-Term Care

**DOI:** 10.1093/geroni/igab046.2724

**Published:** 2021-12-17

**Authors:** Emma King, Alaine Reschke-Hernandez

**Affiliations:** University of Kentucky, Lexington, Kentucky, United States

## Abstract

Growth of the aging population warrants a commitment to teaching students to work effectively with older adults. In an online university course focused on inclusive music practices, students engaged in an extensive service-learning project to enhance course learning objectives and increase sensitivity to disability, social justice, and inclusion issues across the lifespan. Based on community needs, one student aimed to reduce isolation among older adults living in a long-term care facility. The student selected this target and population because of the detrimental effects of isolation on psychosocial, cognitive, and physical well-being; and reduced access to interactive programs and services among older adults living in residential facilities during the COVID-19 pandemic. Components of the project included a service-learning proposal, an annotated bibliography, an interview with the community partner, three activity designs, and a reflective essay. Based on existing research regarding interventions used to reduce isolation among older adults, the three activities the student designed were music bingo, interactive singing, and stretches to music. Throughout the service-learning process, the student consulted the community partner to ensure relevance of the activities. Principles of Universal Design for Learning (UDL), such as providing multiple visual and auditory means to engage with materials, enhanced accessibility of the activities. In this presentation, the student and course instructor will reflect on this case experience and the value of service-learning to foster professional skill development, engaged citizenship in working effectively with older adults, and an appreciation of the need for justice, equity, and fairness for all community members.

